# Folic acid fortification and public health: Report on threshold doses above which unmetabolised folic acid appear in serum

**DOI:** 10.1186/1471-2458-7-41

**Published:** 2007-03-22

**Authors:** Mary Rose Sweeney, Joseph McPartlin, John Scott

**Affiliations:** 1 From the Department of Clinical Medicine, Trinity College, Dublin, Ireland; 2 Department of Biochemistry, Trinity College, Dublin, Ireland; 3 UCD School of Public Health Population Science, University College Dublin, Dublin, Ireland

## Abstract

**Background:**

All flour in the USA is fortified with folic acid at a level of 140 μg/100 g which is estimated to supply an extra 100 μg daily to the average diet. Some researchers have advocated that this be increased to double and even four times this amount. Based on previous research these higher levels are likely to lead to the appearance of unmetabolised vitamin in the circulation, which may have safety implications for sub-groups of the population. The UK and the Republic of Ireland will likely introduce mandatory fortification also in the next year or so.

The aim of this study was to capture the short-term effect of folic acid fortification on unmetabolised folic acid in serum after chronic consumption of folic acid.

**Methods:**

After pre-saturation with 400 μg folic acid supplements daily for 14-weeks, healthy folate replete adults (n = 20) consumed folic acid fortified bread, at three different levels (400 μg, 200 μg, 100 μg) over a period of one week each. The dose was administered in two-equal sized slices consumed at 09.00 hrs and 13.00 hrs. Serum samples for total folate and folic acid were collected at baseline, after 14-weeks of supplementation, and pre and post (at 1, 2, 3 and 4 hours) each dose tested.

**Results:**

Unmetabolised folic acid was detected after the 14-week supplementation period. Folic acid was not detected in either the 200 μg or 100 μg (current US regime) doses tested but was present at the highest level (400 μg) tested.

**Conclusion:**

Our findings suggest that persons exposed to the current US fortification programme supplying an average of 100 μg per day or less are unlikely to have unmetabolised folic acid in serum. It also seems that daily consumption of the higher level of 200 μg or less is unlikely to be problematic. Increasing the level however to 400 μg on the other hand is likely to lead to unmetabolised folic acid appearance.

## Background

To prevent Neural Tube Defects (NTDs) the Food and Drug Administration (FDA), USA implemented mandatory folic acid fortification of flour. This commenced in January 1998. The fortification level was set at 140 μg/100 g flour supplying ~100 μg daily to the average American. This amount was selected on the basis that it would not result in intakes in excess of 1 mg (upper safe daily limit) by any population group. Doubt has been cast on the actual level of fortification however and recent calculations have shown that the level of folic acid fortification is likely to have been over twice the amount mandated [1] with average intakes ranging from 215 to 240 μg/day.

The Centers for Disease Control (CDC) supported the fortification strategy but because this level fell short of the target to supply 400 μg to all women of childbearing age, they considered increasing the level to 350 μg and even 700 μg/100 g, which would supply approximately 2.5 and 5 times more folic acid respectively [2] The primary concern with these higher levels of fortification is that certain segments of the population will be exposed to amounts of folic acid greater than 1000 μg/day and that this may have adverse effects.

After much deliberation by the relevant authorities in the UK folic acid will be added to bread in the UK within a year. In addition the Republic of Ireland have opted for mandatory fortification, which will be fully implemented in the next year.

Research to date suggests that the enzymatic reduction and methylation of folic acid during its absorption in the intestine or its first pass through the liver is dose-dependent. Hence oral folic acid above certain threshold doses saturates the normal intestinal absorptive mechanisms and results in unmetabolised folic acid in serum as well as the normal metabolite 5-methyltetrahydrofolate. This has been demonstrated at oral doses in the region of 200 μg [3] and 266 μg [4]. In a more recent paper it was shown that repeated consumption of physiological amounts of folic acid lead to the accumulation of unmetabolised folic acid in serum [5]. It has also been demonstrated that passive consumption of folic acid in foodstuffs by pregnant women leads to the appearance of unmetabolised folic acid in foetal cord blood [6]. While these studies examined the acute serum response to folic acid, none have investigated the effect after a prolonged period of exposure to the vitamin.

Folic acid has the potential to mask the early haematological manifestations of pernicious anaemia. This has been demonstrated experimentally [7-10] and clinically [11-15]. Other safety considerations of excess folic acid consumption highlighted by the FDA [16] include potential unknown risks for pregnant women, and persons on anti-epileptic and anti-folate medication. The FDA also noted the uncertainties regarding the effects of chronic exposure in children, whose requirements for folate are lower than those of adults. Furthermore evidenced based [17,18] and hypothetical concerns include the potential to promote cancer [19,20] and the recent hypothesis that exposure of the foetus to excess folic acid may favour the selection of the Methylentetrahydrofolate polymorphism, associated with a range of debilitating illnesses [21].

To date no studies have examined the effect of long-term consumption of folic acid on unmetabolised folic acid in serum. The accumulation of folic acid after consumption of fortified bread repeatedly over only one 8-hour period [5] provides justification for this. In this paper we examined unmetabolised folic acid appearance after consumption of folic acid for a prolonged period. The three levels selected to be tested were in the region of that already implemented in the mandatory US fortification programme and the two higher levels considered by the CDC.

## Methods

### Fortification of bread

3 batches of bread were made specifically for the study by Superquinn Bakeries Kimmage, Dublin, under the supervision of the principal researcher. Batch 1 supplied 50 μg per slice, batch 2 supplied 100 μg per slice and batch 3 supplied 200 μg per slice. Each slice weighed about 30 g. The folic acid was added to water, which was then mixed with the flour and other dry ingredients. The dough was formed by thorough mixing with an industrial mixer. The bread was then baked under normal conditions and stored in a food freezer at -20°C until eaten (approximately 2–6 weeks in total). Preliminary research by the researcher showed that folic acid does not degrade under these conditions [22].

### Participants

20 healthy adult subjects, 10 females and 10 males, aged 20–40 years of age were recruited by voluntary participation from the student and staff population of the Central Pathology laboratory, St. James's Hospital, Dublin and from the general population. Ethical approval was obtained from the Ethics Committee of the Federated Dublin Voluntary Hospital, James's Street, Dublin. After informed consent from all participants a medical history was taken and all subjects were found to be free of vascular, hepatic, renal or gastrointestinal disease. Oral contraceptive pill users were excluded from participating, as were those taking multi-vitamin preparations or anti-convulsant therapy. One subject dropped out of the study for personal reasons. 19 subjects completed the study.

#### Intervention-phase I

**Figure 1:**
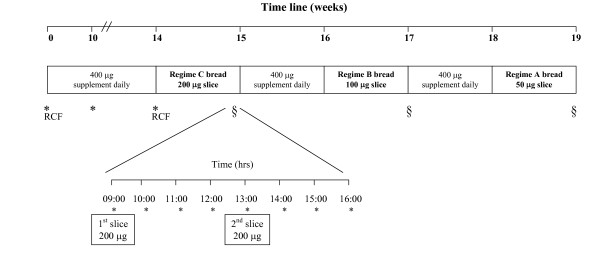
**This flow diagram illustrates diagrammatically the interventions and sampling points**. Schematic summary of intervention phases and sample collection. * = Folate and Folic Acid blood sample. **RCF **= Red cell folate blood sample. **§ **= Day 7, trial of bread regimes (200 μg, 100 μg, 50 μg).

For clarity a flow diagram illustrating diagrammatically the interventions and sampling points is included (figure 1). After confirming that all subjects were folate replete at recruitment by performing red cell and serum folate measurements (table 1) subjects were instructed to consume one 400 μg folic acid supplement daily for 14 weeks. The rationale for this regime was to saturate the subjects at the highest level of fortification to be tested in the study. From previous research [23] we knew it takes 14-weeks to reach a serum folate plateau at this dose. Baseline serum unmetabolised folic acid concentrations were also established (table 1). Subjects were issued with folic acid supplements (Clonfolic 400 μg) and were instructed to omit all other sources of folic acid either in supplemental from or in fortified foods for the duration of the study. A list of commercially available folic acid fortified foodstuffs as well as single/multivitamin preparations containing folic acid on the Irish market was issued to each subject. A list of suitable non-fortified substitutes was issued and subjects were reimbursed financially for these products in order to improve compliance. Subjects were contacted weekly either by telephone or in person throughout the duration of the study to enhance and improve motivation.

**Table 1:** Mean red cell (μg/L) and serum folate (μg/L) concentrations at recruitment (n = 19).

	Serum folate	Red cell folate	Folic acid
Mean	7.60	477.97	0.00
SD	4.25	173.99	-------
CI (95%)	5.55 – 9.65	364.10 – 531.83	-------

This table illustrates mean baseline red cell (μg/L) and total serum folate concentrations (μg/L) at recruitment (n = 19). No subjects had detectable serum folic acid at baseline (>0.312 μg/L).

**Normal reference range **for red cell folate is in the range of 150–1000 μg/L and for total serum folate is 2.7–20 μg/L

Serum samples were collected for red cell, serum folate and unmetabolised folic acid at week 10 and week 14 post-supplementation. At both of these time-points samples were collected 6-hours after the vitamin had last been consumed to allow time for the acute post-prandial folic acid to clear from the circulation.

#### Intervention-phase II

##### Week 1

After the 14-week supplementation period bread was administered to subjects (*Regime C*) supplying a total of 400 μg of folic acid in 2 slices over a period of 7 days. The 1^st ^slice was consumed each day at breakfast time (09.00 hrs) and the 2^nd ^slice was consumed 4 hours later at lunchtime (13.00 hrs).

##### Week 2

Bread was administered to subjects (*Regime B*) supplying a total of 200 μg of folic acid in 2 slices over a period of 7 days. The 1^st ^slice was consumed each day at breakfast time (09.00 hrs) and the 2^nd ^slice was consumed 4 hours later at lunchtime (13.00 hrs).

##### Week 3

Bread was administered to subjects (*Regime A*) supplying a total of 100 μg of folic acid in 2 slices over a period of 7 days. The 1^st ^slice was consumed each day at breakfast time (09.00 hrs) and the 2^nd ^slice was consumed 4 hours later at lunchtime (13.00 hrs).

Between weeks 1, 2 and 3 subjects consumed a 400 μg supplement daily for 7-days in an attempt to re-saturate total serum folate levels.

### Blood collection

On day 7 of each bread regime (A, B and C) the subjects were asked to come to the laboratory at 8 am having fasted from the folic acid fortified bread since the previous lunchtime. Hence approximately 20 hours had lapsed since their last intake of folic acid. An intravenous cannula was inserted and a pre-prandial serum sample (i) was collected for unmetabolised folic acid and serum folate from each subject. This sample was collected in order to establish a pre-prandial baseline before each of the bread regimens. The first slice of bread was then administered. Serum samples were collected every hour for 4 hours (samples ii, iii, iv and v). The second slice of bread was then administered and serum samples were collected every hour for 3 hours (samples vi, vii and viii). It was thought that this pattern would give a good overall picture of the appearance (if present) of unmetabolised folic acid and of the duration of its presence.

### Laboratory analysis

All samples were assayed for total serum folate and red cell folates by the *L. casei *microbiological assay described by [24]. Analysis of samples for unmetabolised folic acid was carried out by HPLC separation of the folic acid followed by solid phase extraction [4]. The re-suspended folic acid was quantified by *the L. casei *microbiological assay above. The lower limit of detection of the assay was calculated to be 0.3125 ng/ml.

### Statistical Analysis

Statistical analysis was performed using the computer package Data Desk 5.0. Repeated measures anova was used when comparing serum folic acid/total folate concentrations at one time point with serum folic acid/total folate concentrations at another time point. Post Hoc Scheffe tests were employed to compare serum folic acid/total folate concentrations at multiple sampling points.

## Results

### Intervention phase 1

Baseline red cell and serum folate concentrations were within normal reference ranges for all subjects (Table 1). Unmetabolised folic acid was not detected in any of the baseline samples collected at week 0 (Table 1). Serum folate concentrations increased significantly from week 0 to week 14 after the supplementation regime (p < 0.001)) as did red cell folate concentrations (p < 0.001) (table 2).

**Table 2:** Mean red cell folate (μg/L), total serum folate (μg/L) and unmetabolised folic acid (μg/L) concentrations at week-14 (n = 19).

Subjects	Serum folate	Red cell folate	Folic acid
Mean	14.77	693.94	0.21
SD	5.01	198.72	0.16
CI (95%)	12.38 – 19.22	434.87 – 903.49	0.29 – 0.52

This table illustrates mean red cell folate (μg/L), total serum folate and unmetabolised folic acid (μg/L) concentrations after consuming a 400 μg folic acid supplement daily for 14 weeks (n = 19). 6 subjects had detectable folic acid levels (>0.312 μg/L).

**Normal reference range **for red cell folate is in the range of 150–1000 μg/L and for total serum folate is 2.7–20 μg/L

At week 14, 6-hours after consumption of the 400 μg supplement unmetabolised folic acid was present in the sera of 18 subjects (n = 19). 7 of these 18 subjects demonstrated only slightly elevated folic acid levels, which were calculated to be below the level of that which is accurately quantifiable. Nonetheless the difference between week 0 and week 14 was statistically significant (p < 0.001) demonstrating that the supplementation did give rise to an elevated folic acid level, which was still detectable six hours after it had been consumed (Table 2).

### Intervention- phase II

**Figure 2:**
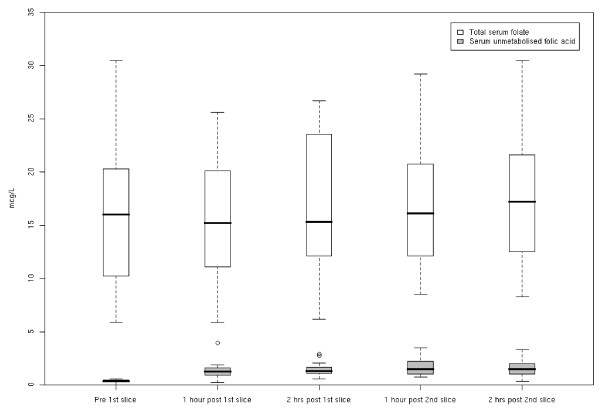
**Mean total serum folate (μg/L) and mean serum unmetabolised folic acid (μg/L) pre and post regime C bread (n = 19)**. This graph illustrates mean total serum folate (μg/L) and mean serum unmetabolised folic acid (μg/L) concentrations in a group of healthy adult subject (n = 19) exposed to a folic fortified bread for 1-week. Subject were pre-saturated with folic acid supplements for 14-weeks prior to the bread. The bread was fortified with 200 μg folic acid per slice, which was administered twice a day for seven days. The samples shown here were collected on the 7^th ^day of bread consumption pre-prandially and post-prandially.

Unmetabolised folic acid was present in the sera of 17 subjects (n = 19) on day 7 of regime C bread pre-prandially (sample i). All of these were above the lowest limit of quantification and were statistically different from week 14 levels (p < 0.001) (see figure 2). Unmetabolised folic acid levels increased post-prandially in all subjects after consumption of the first slice of bread containing 200 μg folic acid (p < 0.001) (figure 2). It was also present in the sera of all subjects after the 2^nd ^slice containing a further 200 μg (p < 0.001) (figure 2).

Post hoc analysis showed that differences in serum folic acid were significant between samples i and samples ii (p < 0.001), samples i and samples iii (p < 0.001), samples i and samples vi (p < 0.001) and samples i and samples vii (p < 0.001). An accumulative elevation of unmetabolised folic acid was demonstrated in 12 out of 19 subjects after the 2^nd ^slice (see figure 2). Unmetabolised folic acid was not detected in the sera of any of the subjects on day 7 of regime B or A bread pre- or post-prandially (tables 3 and 4).

**Table 3:** Serum folate and serum folic acid (μg/l). Samples collected on day 7 of *regime B bread *(100 μg in each slice) pre and post prandially.

	pre 1^st ^slice	1 hour post	2 hrs post	3 hrs post	4 hrs post	1 hour post 2^nd ^slice	2 hrs post	3 hrs post
Serum folate	i	ii	iii	iv	v	vi	vii	viii
Mean	14.1111	14.70	15.16	14.72	14.41	14.43	13.84	13.28
SD	5.24	5.59	4.99	5.32	5.83	5.78	5.25	5.47
CI (95%)	11.59 – 16.63	12.00 – 17.39	12.75 – 17.56	12.16 – 17.28	11.60 – 17.22	11.65 – 17.22	11.23 – 16.45	10.46 – 16.10

This table illustrates mean total serum folate (μg/L) concentrations in a group of healthy adult subject (n = 19) exposed to a folic fortified bread for 1-week. Subject were pre-saturated with folic acid supplements for 14-weeks prior to the bread. The bread was fortified with 100 μg folic acid per slice, which was administered twice a day for seven days. The samples shown here were collected on the 7^th ^day of bread consumption pre-prandially and post-prandially. Standard deviations and confidence intervals are also shown. Serum folic acid was not detectable at any sampling points

**Table 4:** Serum folate and folic acid (μg/L). Samples collected on day 7 of regime A bread (50 μg in each slice) pre and post prandially.

	pre 50 μg	1 hour post	2 hrs post	3 hrs post	4 hrs post	1 hour post 2^nd ^slice	2 hrs post	3 hrs post
Serum folate	i	ii	iii	iv	v	vi	vii	viii
Mean	16.91	16.30	16.99	16.88	17.40	15.56	15.40	14.82
SD	6.55	6.62	6.42	6.24	7.12	5.77	6.04	5.21
CI (95%)	13.75 – 20.06	13.11 – 19.49	13.89 – 20.08	13.87 – 19.88	13.97 – 20.83	12.78 – 18.34	12.49 – 18.31	12.31 – 17.33

This table illustrates mean total serum folate (μg/L) concentrations in a group of healthy adult subject (n = 19) exposed to a folic fortified bread for 1-week. Subject were pre-saturated with folic acid supplements for 14-weeks prior to the bread. The bread was fortified with 50 μg folic acid per slice, which was administered twice a day for seven days. The samples shown here were collected on the 7^th ^day of bread consumption pre-prandially and post-prandially. Standard deviations and confidence intervals are also shown. Serum folic acid was not detectable at any sampling points

Total folate concentrations did not change significantly over the 8 hours after regime C (P = 0.8941), regime B (P = 0.9820) or regime A bread (p = 0.8829) (figure 2).

## Discussion

Unmetabolised folic acid was not detected in any of the subjects at week 0. It was detected in the serum of all subjects after consumption of a 400 μg folic acid supplement daily for 14 weeks even though the samples were collected six-hours after the last supplement. It is likely then that women complying with the current DOH (UK) [25] recommendation to consume a 400 μg supplement daily for their entire childbearing years would be exposed to unmetabolised folic acid in serum for at least six out of twenty-four hours every day. The safety implications of this are unknown but long-term surveillance of serum folic acid at a population level may be useful to observe if an accumulation occurs over time.

Out of the three fortified bread regimens administered only the highest one (400 μg administered in two equal doses) lead to the appearance of folic acid in serum. Both the first and the second slice lead to this effect and an accumulation was noted after the second one. Whether these levels would accumulate indefinitely or plateau at some point with prolonged repeated exposure in a population exposed to these levels under a fortification programme is unknown but again ongoing surveillance is indicated.

From a positive viewpoint our data shows that unmetabolised folic acid did not appear in serum after consumption of the first or second slice of bread containing either 50 μg or 100 μg each over a period of 1 week even after a period of pre-saturation. The latter is the amount currently supplied to the average US citizen in the US fortification programme. It is important to remember however that the level of fortification currently in place in the USA is based on FDA calculations for delivery of 100 μg of folic acid at "average intakes". Folic acid in the diet from other sources other than mandatory folic acid fortified bread will of course augment these intakes, as breakfast cereals and many other foodstuffs in the United States are also fortified with folic acid, for example consumption of a couple of servings of ready-to-eat breakfast cereals and toast would easily exceed 200 μg in a single dose. In addition persons complying with the recommendation to consume a 400 μg supplement daily (estimated to be 30 – 40% of Americans) will have sufficient intakes to lead to unmetabolised folic acid in serum before they even consume any fortified foods.

## Conclusion

Based on the cumulative evidence to date in this and other studies [4-6] it seems that the threshold dose above which unmetabolised folic acid appears in serum lies around 200 μg. The intervals between repeated exposures also seem to be an important factor [6] as an accumulative effect is observed if repeated doses are consumed close together. These are important considerations for policy makers planning an intervention.

## Competing interests

There are no conflicts of interest for any of the authors in relation to this work. The lead authors had full access to all the data in the study and had final responsibility for the decision to submit for publication.

## Authors' contributions

MRS designed and conducted the research study (both the nutrition intervention phase and the laboratory work). She also analysed and interpreted the data and drafted the manuscript.

JS had the original idea for the study and advised on all aspects of the study.

JMcP advised and assisted with all phases of the project

All authors read and approved the final manuscript.

## Pre-publication history

The pre-publication history for this paper can be accessed here:


